# Rooting binder-free tin nanoarrays into copper substrate via tin-copper alloying for robust energy storage

**DOI:** 10.1038/s41467-020-15045-x

**Published:** 2020-03-05

**Authors:** Jiangfeng Ni, Xiaocui Zhu, Yifei Yuan, Zhenzhu Wang, Yingbo Li, Lu Ma, Alvin Dai, Matthew Li, Tianpin Wu, Reza Shahbazian-Yassar, Jun Lu, Liang Li

**Affiliations:** 1 School of Physical Science and Technology, Center for Energy Conversion Materials & Physics (CECMP), Soochow University, 215006 Suzhou, P. R. China; 2 Chemical Sciences and Engineering Division, Argonne National Laboratory, 9700 South Cass Avenue, Lemont, IL 60439 USA; 3 Mechanical and Industrial Engineering Department, University of Illinois at Chicago, Chicago, IL 60607 USA; 4 Key Laboratory of Advanced Ceramics and Machining Technology (Ministry of Education), School of Materials Science and Engineering, Tianjin University, 300072 Tianjin, China; 5 Advanced Photon Sources, X-ray Science Division, Argonne National Laboratory, 9700 South Cass Avenue, Lemont, IL 60439 USA

**Keywords:** Energy science and technology, Batteries

## Abstract

The need for high-energy batteries has driven the development of binder-free electrode architectures. However, the weak bonding between the electrode particles and the current collector cannot withstand the severe volume change of active materials upon battery cycling, which largely limit the large-scale application of such electrodes. Using tin nanoarrays electrochemically deposited on copper substrate as an example, here we demonstrate a strategy of strengthening the connection between electrode and current collector by thermally alloying tin and copper at their interface. The locally formed tin-copper alloys are electron-conductive and meanwhile electrochemically inactive, working as an ideal “glue” robustly bridging tin and copper to survive harsh cycling conditions in sodium ion batteries. The working mechanism of the alloy “glue” is further characterized through a combination of electrochemical impedance spectroscopy, atomic structural analysis and in situ X-ray diffraction, presenting itself as a promising strategy for engineering binder-free electrode with endurable performance.

## Abstract (web summary)

The authors here report a binder-free electrode based on tin nanoarrays deposited on copper substrate. It is found that the locally formed electrochemically inactive tin-copper alloys work as a glue that bridges tin and copper to survive harsh cycling conditions in sodium ion batteries.

## Introduction

A tight and reliable bonding between electrode materials and current collectors is the prerequisite toward high-rate and endurable energy storage property in rechargeable ion batteries^1^. Most strategies addressing this issue involve the use of polymer-based binders, such as PVDF, which, although efficiently bond electrode particles with each other to the current collectors, severely decrease the energy density due to their electrochemical inactivity^2^. What’s worse, the electron-insulating nature of binders cuts off the electron pathway among particles and thus degrades the rate performance of associated electrode materials.

As such, binder-free electrodes have been developed to push the energy density limit of active battery materials by increasing their ratio in the electrodes^3^. Some methods such as electrochemical deposition have been reported to successfully prepare binder-free electrode with the active materials tightly grown to the surface of current collector^4,5^. In spite of the achieved high energy density of these designs, the long-term cycling stability is significantly compromised due to the lack of efficient binding intermediates^6^. This situation is worsened for electrode materials experiencing intrinsic volume expansion and contraction during repetitive battery cycling, such as transitional metal oxide with a conversion-based energy storage mechanism and silicon (tin, germanium, etc.) with an alloying-based mechanism^7^.

For example, Sn, as a sodium ion battery anode, features a theoretical capacity of 847 mAh g^−1^ (Sn → Na_15_Sn_4_) with high electronic conductivity, low toxicity and low sodiation potential of ~0.3 V (vs. Na^+^/Na). However, the formation of Na_15_Sn_4_ alloy results in a huge volume expansion of 420%, which leads to the generation of fractures and cracks inside Sn electrodes and continuous evolution of solid electrode/electrolyte interphase (SEI)^8^. To mitigate these issues, several structural designs on Sn anodes have been established in recent years. One representative design involves the synthesis of composites of ultrafine Sn nanoparticles encapsulated in carbon matrix, which, however, only provides moderate specific capacity due to the presence of dense carbon^9,10^. Another approach refers to three-dimensional (3D) electrode architectures based on templates, for example, virus or wood^11,12^. Architectured electrodes exhibit enhanced energy storage performance due to the unique structural characteristics of large accessible surface, free electrolyte permeation channel, additional space for volume change^6,13,14^. Specifically, nanowalls are particularly useful due to the shorter diffusion distance for Na-ions to reach the core of the metal nanowalls. Furthermore, the space between nanowalls allows the free expansion of Sn electrodes to release stress and strain, thus minimizing the possibility of electrode failure. However, the key problem associated with deposited arrays of nano-sized dimensions is their adhesion to the substrate. Over cycling, the detachment of Sn electrode from the substrate/current collector is inevitable due to the continuous volume expansion/contraction, regardless of whether implementing binders or binder-free strategy.

Targeting Sn as an anode for sodium-ion batteries, we demonstrate here a strategy of strengthening the connection between the electrode (Sn) and the current collector (Cu) by thermally alloying Sn and Cu at their interface region. Sn nanowall-shaped arrays (SnNA) are first electrochemically deposited onto Cu substrate, which is then followed by a mild (180 °C) but key heat treatment step. The presence of electrochemically inactive Sn–Cu alloy serves as a structural glue to guarantee the adhesion between SnNA and Cu substrate. More importantly, the gradient-like distribution of Sn–Cu ensures no abrupt change in volume expansion/contraction during repetitive sodiation/desodiation cycles and therefore maintains the overall structural integrity over long cycles. When directly used as an electrode for sodium storage, SnNA exhibits a reversible capacity of 801 mAh g^−1^ at 0.2 C (where C is referred to as the current for full charge or discharge of the theoretical capacity in 1 h, i.e., 847 mA g^−1^), a rate capability of 610 mAh g^−1^ at 5 C, and a retention of 501 mAh g^−1^ at 5 C after 300 cycles. Considering its remarkable electrochemical performance and simple fabrication with scale-up capability, SnNA tightly rooted into current collector via Cu–Sn alloying mechanism might inspire future engineering of electrode structures foe endurable energy storage applications.

## Results

### Physical characteristics

**Fig. 1:**
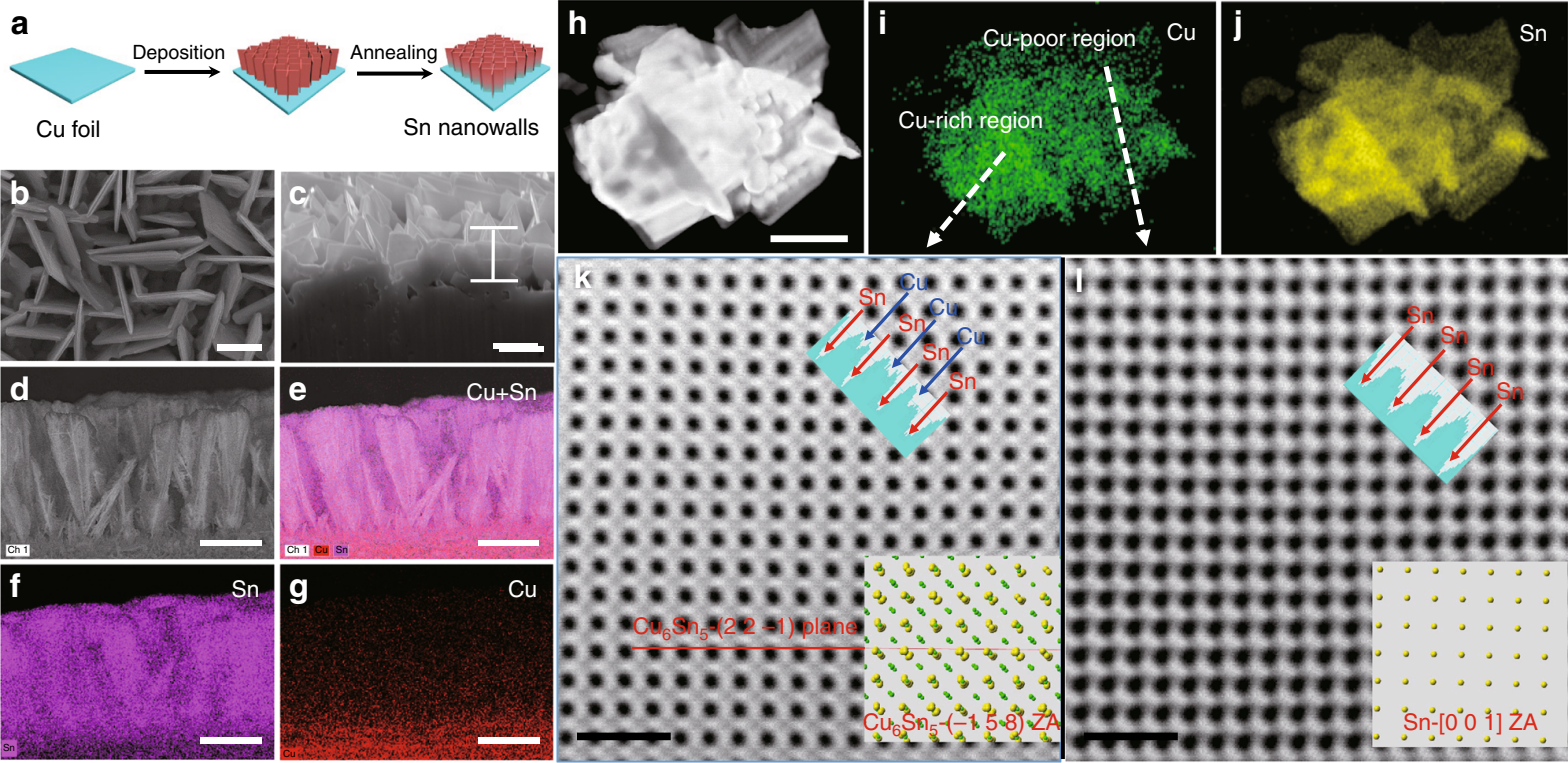
Synthesis, morphology and structure analyses of SnNA. **a** Schematic illustration of electrochemical synthesis process. SEM images of **b** top view and **c** side view. **d**–**g** Elemental mapping of energy dispersive X-ray spectroscopy reveals the diffusion of Cu throughout the nanowall. **e** Cu and Sn, **f** Sn, and **g** Cu. **h** Low-mag STEM-HAADF image of one SnNA. **i**, **j** EDS mapping of Cu (green) and Sn (yellow). **k** STEM-ABF image of the Cu_6_Sn_5_ atomic structure viewed along the zone axis of [−1 5 8]. **l** STEM-ABF image of the Sn atomic structure viewed along the zone axis of [0 0 1]. **k**, **l** The line profiles are given and compared in the insets to highlight the presence of Cu columns in Cu_6_Sn_5_. Atomic models matching each experimental observation are also given in the right bottom insets with yellow sphere for Sn and green sphere for Cu. Scale bar: **b** 1 μm, **c**–**g** 2 μm, **h** 1 μm, **k**, **l** 1 nm.

Aligned Sn nanowalls were electrochemically grown on Cu substrate by a template-free deposition, followed by thermal annealing at 180 °C for 2 h in Ar flow, as illustrated in Fig. 1a. Details of the fabrication can be found in Methods and Supplementary Information. Scanning electron microscopy (SEM) characterization reveals the deposited product as an architecture of interwoven nanowalls vertically grow onto Cu foil (Supplementary Fig. 1). No morphological change of Sn nanowall arrays is observed after thermal annealing at 180 °C in Ar flow. The individual nanowall has a nanosheet structure, with a thickness of 50−100 nm and a height of about 2.6 μm (Fig. 1b, c). We speculate that the growth of Sn nanowalls might be attributed to a glycol-directed assembly^15^. Firstly, glycol-coordinated Sn^4+^ ions will be reduced to metallic Sn driven by current. At the mediation of glycol, the cross growth of Sn species is restricted by the hydrocarbon periphery, while the growth of Sn nanosheets is then preferred (Supplementary Fig. 2). Without the mediation from glycol, only nanoparticles of Sn can be obtained (Supplementary Fig. 3). Interestingly, evident diffusion of Cu element into Sn matrix for alloying reaction is observed throughout the annealed nanowalls via elemental mapping analysis (Fig. 1d–g). This is because Cu-Sn alloy reaction proceeds significantly at temperatures above 100 °C^16^. Since the alloying process is controlled by diffusion, the concentration profile of Cu in the Sn nano wall will naturally increase when one moves closer toward the Cu substrate, as reflected by Fig. 1d–g. This ensures a smooth gradient blend of Sn and Cu species at the alloying region, which is key to regulate the volume variation of Sn upon cycling. Scanning transmission electron microcopy (STEM) imaging further confirms the elemental distribution of Cu and Sn as well as their regional mixing state (Fig. 1h–j). Atomic STEM-ABF images shown in Fig. 1k, l exhibit distinct crystalline patterns from two locations indicated by the white arrows. Compared to Fig. 1l showing pure Sn atomic columns, the Sn atomic columns in Fig. 1k are separated by extra atomic columns of lighter elements. Since Cu is much lighter than Sn, such extra atoms are proposed to be Cu (note that STEM imaging contrast is associated with atomic weight)^17,18^. This is further confirmed by indexing the two atomic patterns in Fig. 1k, l to standard phases of Cu_6_Sn_5_ alloy^19^ and tetragonal Sn^20^, respectively. The formation of alloy phases has also been corroborated by transmission electron microcopy (TEM) shown in Supplementary Fig. 4.

**Fig. 2:**
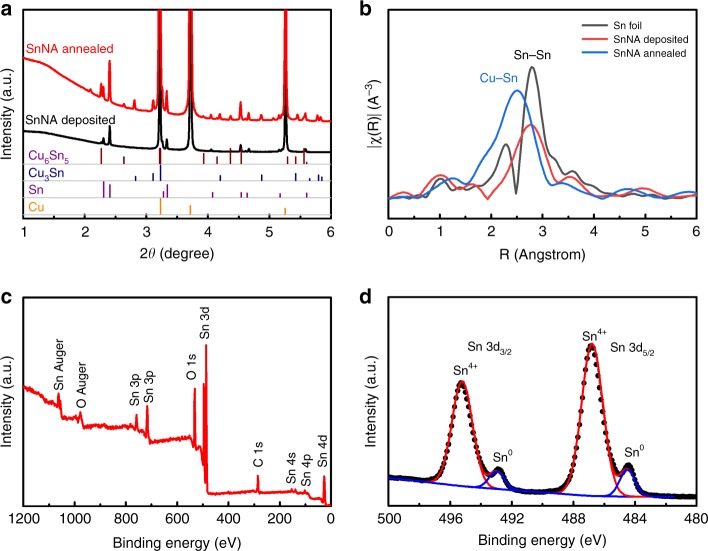
Structural characterization of SnNA. **a** Ex-situ high-energy XRD patterns of as-deposited and annealed SnNA samples, showing the formation of alloy phases of Cu_3_Sn and Cu_6_Sn_5_ after thermal annealing at 180 °C. **b** Fourier transformation curves of XAFS for Sn edges. Sn foil is used as a reference. **c** XPS survey spectrum. **d** XPS core-level spectrum of Sn 3d, showing the presence of surface tin oxides.

Ex-situ high-energy X-ray diffraction (XRD) patterns in Fig. 2a reveal the significant difference between SnNA samples with and without annealing treatment. The deposited Sn sample is well crystallized in a tetragonal phase (PDF #04-0673). After a mild annealing at 180 °C, although tetragonal Sn remains the main phase, new phases related to orthorhombic Cu_3_Sn (PDF #01-1240) and Cu_6_Sn_5_ (PDF #02-0713) appear. The new phases are derived from the alloying process of Sn with Cu, which has been facilitated by heating^16^. To explore the structural evolution and atomic arrangements of SnNA upon thermal annealing, we conducted X-ray absorption (XAS) measurement. We focus on the X-ray absorption fine structure (XAFS) at Sn K-edge, the corresponding Fourier transformation curves of which are depicted in Fig. 2b. The Fourier transformation spectrum of Sn foil exhibits one main peaks at 2.79 Å, corresponding to the Sn–Sn coordination^21^. As-deposited SnNA shows a similar peak at a position of 2.76 Å, which shifts to a remarkably lower location of 2.52 Å after thermal annealing. This lower-location peak suggests the prevailing existence of Sn–Cu bonding in annealed SnNA, whose bond length is distinctly shorter than that of Sn–Sn coordination^22^. This is in good agreement with the result of XRD, and further confirms the generation of alloy phase of Cu_3_Sn and Cu_6_Sn_5_ by mild annealing.

No metal oxides such as SnO and SnO_2_ are detected according to the Raman scattering spectrum of the final SnNA product (Supplementary Fig. 5)^23^. However, by nature, a tin oxide layer would be formed upon exposure of Sn nanowalls to air. The presence of surface oxide layer could be probed by X-ray photoelectron spectroscopy (XPS, Fig. 2c). Notably, there is a C1s signal in the XPS survey spectrum, which is due to ubiquitous carbon contamination on samples during air exposure^24^. Figure 2d presents the core-level spectrum of Sn 3d consisting of two peaks located at 495.2 and 486.7 eV, which can be assigned to Sn^4+^, and two another peaks at 492.8, 484.3 eV attributed to metallic Sn^20^. Despite a stronger signal of Sn^4+^ compared to Sn, the content of tin oxides should be negligible as XPS is a surface technique with a penetration depth within several nanometers.

### Electrochemical properties

**Fig. 3:**
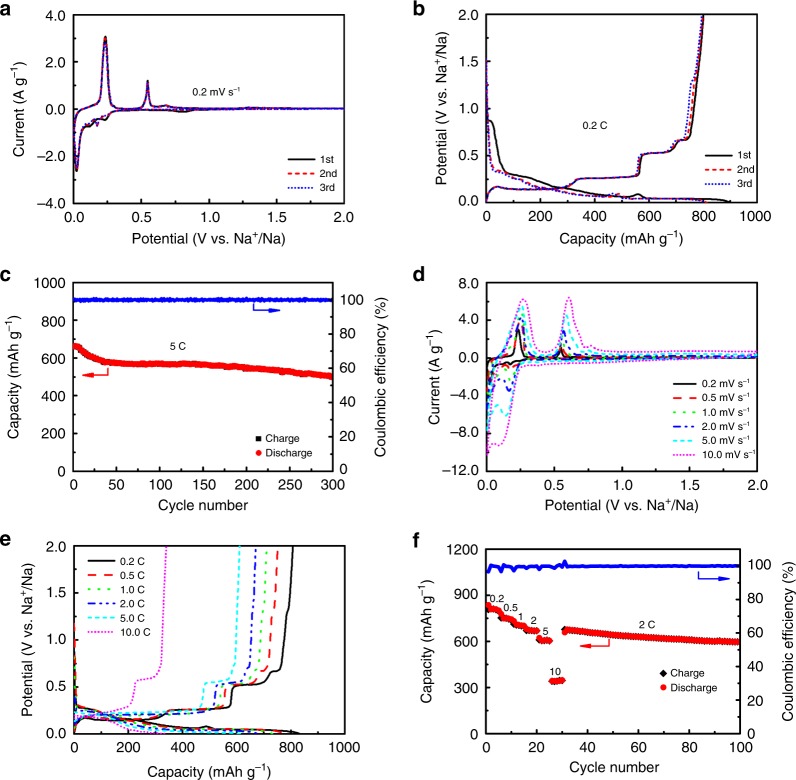
Electrochemical sodium storage in SnNA. **a** CV curve at a sweep rate 0.2 mV s^−1^ for initial three cycles. **b** Galvanostatic curves at a rate of 0.2 C during initial three cycles. **c** Cycling performance at a high charge-discharge rate of 5 C for 300 cycles. The cell is initially activated at a low rate of 0.2 C for 5 cycle. **d** CV curves at various sweep rates ranging from 0.2 to 10 mV s^−1^. **e** Galvanostatic charge-discharge curves at current rates ranging from 0.2 to 10 C. **f** Rate cycling performance first at various current rates and then at a fixed rate of 2 C.

To demonstrate the superiority of such unique architecture of SnNA, electrochemical sodium coin-type cells were assembled and tested. A piece of annealed SnNA film was directly used as the working electrode, a Na foil as the counter electrode, and 1 M NaPF_6_ dissolved in diglyme as the electrolyte. Electrochemical behaviors of SnNA were examined by cyclic voltammetry (CV) and galvanostatic charge and discharge, as presented in Fig. 3. The initial CV curve at a scan rate of 0.2 mV s^−1^ shows an evident peak at 0.80 V, which diminishes at the following cycles, characteristic of the formation of SEI. Three redox peaks at 0.23, 0.12, and 0.03 V represent a stepwise alloying of Sn with Na to form NaSn, Na_9_Sn_4_, and Na_15_Sn_4_, respectively^25^. The corresponding dealloying peaks occur at 0.24, 0.55, and 0.68 V. The stepwise (de)alloying process is further confirmed by galvanostatic test at 0.2 C (Fig. 3b). SnNA exhibits an initial sodiation capacity of 898 mAh g^−1^, out of which a capacity of 801 mAh g^−1^ is reversibly desodiated. The Coulombic efficiency (89%) in the first cycle could be further enhanced to 92% by annealing SnNA in Ar/H_2_ (*v*/*v* = 95:5) flow to eliminate trace oxides, which are less electrochemically reversible (Supplementary Fig. 6). Besides possessing a high capacity, SnNA also shows a stable cycling (Fig. 3c), retaining a desodiation capacity of 501 mAh g^−1^ (76% of the initial value) over 300 cycles at a high rate of 5 C. In contrast, the electrodeposited product of Sn particles exhibit a much lower desodiation capacity of 567 mAh g^−1^ with a poor cycling stability (Supplementary Fig. 7). The SnNA electrode/electrolyte interphase is monitored by electrochemical impedance spectroscopy. The almost unchanged spectra of electrode upon 100 cycles (Supplementary Fig. 8) suggest that the nanoglue (Sn–Cu alloy) is indeed quite stable. Moreover, post-mortem SEM and TEM images in Supplementary Fig. 9 confirms the conservation of nanowall structure after long-term cycles of Na reaction.

Kinetic characters of SnNA were explored further by CV at various sweep rates ranging from 0.2 to 20.0 mV s^−1^ (Fig. 3d and Supplementary Fig. 10). Impressively, the CV curve well maintains the shape and position of redox peaks at each sweep rate, signifying a robust kinetic behavior. Similarly, galvanostatic curves shows a vigorous charge-discharge rate capability (Fig. 3e). SnNA affords desodiation capacities 807, 752, 712, 671, and 610 mAh g^−1^ at rates of 0.2, 0.5, 1, 2, and 5 C, respectively. Even at a much higher rate of 10 C, it still affords a reversible capacity of 341 mAh g^−1^. Despite a decrease in capacity upon increasing rates, the potential plateaus and the small polarization are well preserved. High-rate stability and reversibility are further corroborated in Fig. 3f. Such a 3D SnNA electrode is superior to many Sn-based electrodes (Supplementary Table 1)^9–12,26–28^, unambiguously proving the efficacy of our electrode design.

### Mechanism analysis

**Fig. 4:**
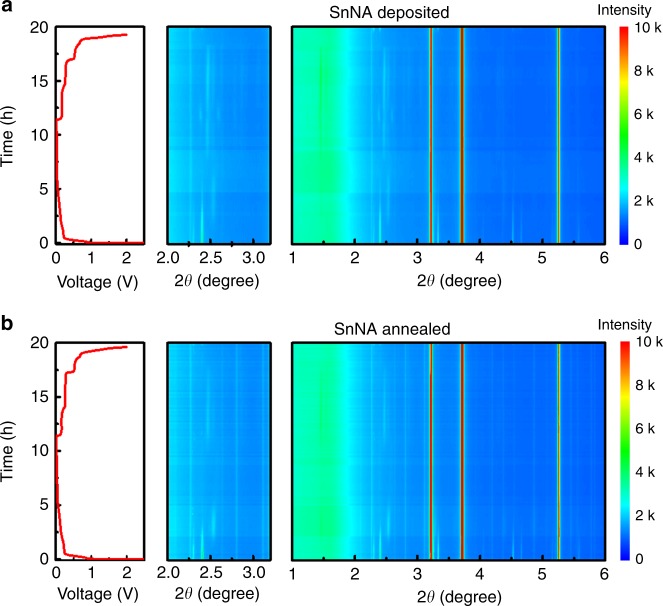
Structure evolution of SnNA electrode in a working coin cell with Na metal as the counter revealed by in-situ XRD. **a** As-deposited SnNA, **b** As-annealed SnNA. **a**, **b** The left panel is the voltage profile of the electrode, while the right is the two-dimensional diffraction patterns collected in a transmission mode with an exposure time of 60 s. Intermediate crystalline phases of Na_9_Sn_4_ (peaks at 1.462 and 2.530°) and Na_15_Sn_4_ (peaks at 2.392 and 2.602°) are clearly observed for sodiated SnNA electrodes. The middle panel is a zoomed pattern where Cu_3_Sn and Cu_6_Sn_5_ alloy phases remain intact upon (de)sodiation.

As shown previously in our microscopy and post-cycling EIS studies, it is clear that there are no apparent changes in both the morphology and electron/ionic conduction pathways. To track the structural evolution during cycling, in situ high-energy XRD experiments were conducted. Figure 4a, b show the two-dimensional contour plots for the local structural and phase evolution of both deposited and annealed SnNA electrodes during the 1st galvanostatic cycle at 0.1 C, where red color represents high intensity while blue color stands for low intensity. As expected, at the beginning of sodiation, the diffraction peaks of Sn gradually weaken, while an intermediate phase of Na_9_Sn_4_ (PDF #31-1326) with characteristic peaks at 1.462° and 2.530° appears for both samples. Upon the further sodiation, these peaks gradually decrease in intensity, and in particular the peak at 2.530° shifts negatively to 2.465°. Concurrently, new diffraction peaks at 1.447°, 1.608°, 2.392°, and 2.602° characteristic of crystalline Na_15_Sn_4_ (PDF #31-1327) tardily appears, indicating a further alloying process. At the final discharge state of 1 mV, the characteristic peaks of Na_9_Sn_4_ and Na_15_Sn_4_ remain visible, suggesting a co-existence between the different phases^29^. Additionally, the peaks corresponding to Na_9_Sn_4_ and Na_15_Sn_4_ disappeared while the Sn XRD peaks could return to their original position once charged back to 2 V, indicating that the (de)sodiation process is highly reversible. It is worth mentioning that the peaks due to phases of Cu_6_Sn_5_ and Cu_3_Sn derived from annealing remain intact upon sodium cycling, suggesting that the formation of Na–Cu–Sn alloy is electrochemically unfavorable. Recent studies reveal that Cu–Sn alloys exhibit a much lower activity toward sodium than lithium, and their sodiation process is quite similar to that of the pure Sn^30^. This unusual behavior suggests the sodiation activity might come from the aggregation of Sn rather than the other part of Cu–Sn alloys. This means that these nanoglue phases are electrochemically inert and not prone to stresses experienced from the sodiation/desodiation process^31^, which can further prevent electrode cracks or any material disconnections with the current collector caused by volume variation of Sn. Following this observation, it is clear that the volume expansion of the Cu–Sn alloy/Sn blends will depend on their composition. We believe, due to the concentration gradient produced from a diffusion-based Cu–Sn alloying process, the volume expansive stress also decreases in a smooth manner (non-abrupt) as one moves from the pure Sn nanowalls toward the pure Cu substrate. This concentration gradient profile has been verified by the XPS spectra collected at different etching depths (durations) by Ar^+^ ion, as shown in Supplementary Fig. 11, where the content of Cu continuously increases from the top Sn to the bottom substrate. As Supplementary Fig. 12 shows, SnNA without thermal annealing only retains 326 mAh g^−1^, or 70% of its initial value over 100 cycles at 2 C, which is much inferior to the annealed counterpart.

**Fig. 5:**
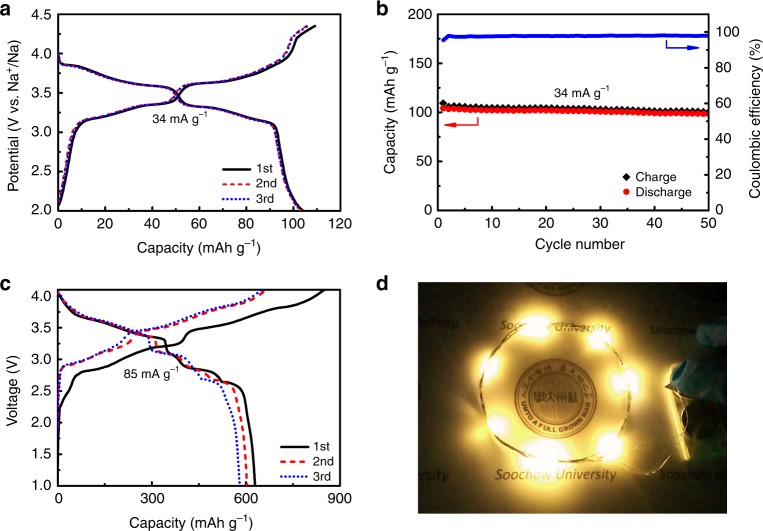
Electrochemical performance of SnNA//Na_0.67_(Ni_0.23_Mg_0.1_Mn_0.67_)O_2_ full cell. **a**, **b** Electrochemical performance of Na_0.67_(Ni_0.23_Mg_0.1_Mn_0.67_)O_2_ cathode, **a** galvanostatic charge-discharge curves and **b** cycling performance at a rate of 34 mA g^−1^. **c** Galvanostatic curves of SnNA//Na_0.67_(Ni_0.23_Mg_0.1_Mn_0.67_)O_2_ full cell at a rate of 85 mA g^−1^. The capacity is calculated based on the mass of SnNA anode. **d** Photograph shows two charged cells in series lighting up LED arrays.

In light of its remarkable electrochemical activity and durability enabled by the unique 3D architecture, we evaluate the potential of SnNA electrodes in practical reality by assembling full cells of SnNA//Na_0.67_(Ni_0.23_Mg_0.1_Mn_0.67_)O_2_. To minimize irreversible capacity loss, the mass ratio between anode and cathode is carefully adjusted to 1:8. The Na_0.67_(Ni_0.23_Mg_0.1_Mn_0.67_)O_2_ cathode was prepared via a sol–gel approach^32^, followed by a conventional doctor-blade technique. Details of materials and battery assembly can be found in Methods section. As Fig. 5a, b shows, the Na_0.67_(Ni_0.23_Mg_0.1_Mn_0.67_)O_2_ cathode affords a reversible capacity of ∼104 mAh g^−1^ at 34 mA g^−1^ with favorable cycling stability. The full cell of SnNA//Na_0.67_(Ni_0.23_Mg_0.1_Mn_0.67_)O_2_ exhibits a reversible capacity of 629 mAh g^−1^ (based on the anode mass) in the voltage range of 4.1–1.0 V. The average working voltage is ~3.2 V, and the specific energy is calculated to be 199 Wh kg^−1^ based on the mass of active materials. When fully charged, the cell works well in lighting up LED arrays, thereby demonstrating its potential in practical energy application for electronics.

## Discussion

We have demonstrated the template-free construction of 3D Sn nanowall arrays tightly rooted into Cu substrate via Sn–Cu alloying reactions. Our unique mild annealing step, following the Sn electrodeposition onto Cu, led to the formation of Cu–Sn alloy phases, which serves as an adhesive or so-called “nanoglue” between the SnNA and Cu substrate. This nanoglue prevented the disconnection of the SnNA from the Cu substrate over cycles of sodium ion batteries. Our anode design delivered a high reversible capacity of 801 and 610 mAh g^−1^ at 0.2 and 5 C, respectively. Furthermore, we have developed full cells of SnNA//Na_0.67_(Ni_0.23_Mg_0.1_Mn_0.67_)O_2_ capable of affording a working voltage of ~3.2 V and a specific energy of 199 mWh g^−1^ (on the basis of active materials). The strategy of developing and consolidating 3D binder-free electrodes is applicable to other alloy-based and conversion-based electrodes that have been plagued by the notorious cycling-induced volume variation, and hence may pave the way for the development of high-capacity and high-rate anodes in various rechargeable battery systems.

## Methods

### Synthesize of SnNA

Sn nanowall array was directly deposition on Cu foil (0.1 mm, 99.95%) in a glycol/H_2_O (1:1 by volume) electrolyte consisting of 0.06 M SnCl_4_ and 0.12 M trisodium citrate. The electrodeposition employs a three-electrode set-up with a piece of Cu foil as the working electrode, a platinum gauze as the counter electrode, and a Ag/AgCl as the reference electrode. The electrodeposition was carried out at a current of 0.01 A cm^−2^ for 1200 s with a constant stirring of 100 rotations min^−1^ at ambient temperature (~25 °C). Prior to deposition, Cu substrates were ultrasonically cleaned in ethanol, diluted acetic acid and ethanol successively. One side of Cu foil is covered by scotch tape to ensure the deposition only occurring in the other side. The deposited Sn nanowall array was rinsed with water and ethanol, dried at 100 °C in vacuum, and finally annealed in Ar flow at 180 °C for 2 h. The mass of SnNA was estimated to be 0.6 mg cm^−2^ by measuring the weight difference before and after deposition. By contrast, Sn thin film rather than nanowall was deposition in aqueous electrolyte of 0.1 M SnCl_4_ without glycol.

### Characterization

Morphology of SnNA samples were characterized by SEM (Hitachi SU-8010) and TEM (FEI Tecnai G2 T20). The structure of SnNA samples was characterized by a Rigaku D/MAX-2000PC XRD diffractometer and a HORIBA Jobin Yvon LabRAM HR800 Raman spectroscopy. Chemical composition of samples was analyzed by XPS (Thermo Fisher Scientific Escalab 250Xi). An Al anode running at 250 W was used as the X-ray source and the base pressure in the analytic chamber about 5 × 10^−9^ Torr. The atomic imaging is done using an aberration-corrected STEM (JEOL ARM200CF) operated at 200 kV.

### X-ray absorption spectroscopy (XAS) measurements

Ex-situ of XAS of the as-prepared SnNA without and with thermal annealing electrodes were measured at 20-BM-B of APS at Argonne National Laboratory in transmission mode with electron energy of 7 GeV. Sn K-edge spectra were collected and processed by Athena software.

### Ex-situ and in-situ high-energy X-ray diffraction (XRD) measurements

Ex-situ and in-situ XRD of the as-prepared SnNA electrodes without and with thermal annealing were measured at 11-ID-C of the Advanced Photo Source at Argonne National Laboratory with a wavelength of 0.1173 Å. Two-dimensional diffraction patterns were collected in a transmission mode with an exposure time of 60 s. A standard sample of CeO_2_ was applied to calibrate the 2D diffraction patterns, which can be converted to one-dimensional patterns using Fit 2D software. For the in-situ XRD experiments, home-made coin cells and Na foil anode with 3 mm holes were used to allow beam pass through. The holes at the both sides of cases of coin cell were sealed with Kapton tape before cell assembly. During the in situ XRD study, the cells were discharged and charged at a constant rate of 0.1 or 0.2 C between 1 mV and 2 V via a MACCOR cycler. 2D diffraction patterns were recorded around every 600 s, which could capture the structural and phase evolution during the 1st cycle of cell.

### Electrochemical tests

The electrochemical evaluation was performed on 2032-type coin cells assembled in an Ar-filled Mikrouna glove box. The half-cell consists of a SnNA working electrode and a Na foil counter electrode. Galvanostatic tests of the cells were operated on a Land CT2001A battery test system. Cyclic voltammetry and electrochemical impedance spectroscopy were performed on an Autolab electrochemical workstation. All electrochemical tests were performed at room temperature (~25 °C). To assemble full cells, Na_0.67_(Ni_0.23_Mg_0.1_Mn_0.67_)O_2_ cathode material was used. The Na_0.67_(Ni_0.23_Mg_0.1_Mn_0.67_)O_2_ material was synthesized by a sol–gel route. A mixed aqueous solution of sodium, nickel, magnesium and manganese and citric acid in a stoichiometric ratio was bathed at 60 °C under continuous stirring until most water was evaporated. The resulting gel was then decomposed at 400 °C and finally annealed at 900 °C for 15 h in air. To make cathode slurry, 80% of cathode material, 5% carbon black, 5% carbon nanotube, and 10% polyvinylidene fluoride were homogeneously mixed and cast onto an Al foil. The electrode sheet was cut into disks with a diameter of 12 mm. The electrolyte is 1 M NaPF_6_ dissolved in diglyme.

## Supplementary information

Supplementary Information

## Data Availability

The data that support the findings of this study are available from the corresponding author upon reasonable request.
