# Supplementary material for: Rooting binder-free tin nanoarrays into copper substrate via tin-copper alloying for robust energy storage
Source: Nat Commun. 2020 Mar 5;11:1212. doi: 10.1038/s41467-020-15045-x (PMC7058056; doi:10.1038/s41467-020-15045-x)
Supplement: Supplementary file 1 — Supplementary Information [file 41467_2020_15045_MOESM1_ESM.pdf]

Supplementary Information

Rooting binder-free tin nanoarrays into copper substrate *via* tin-copper alloying for robust energy storage

Ni et al.

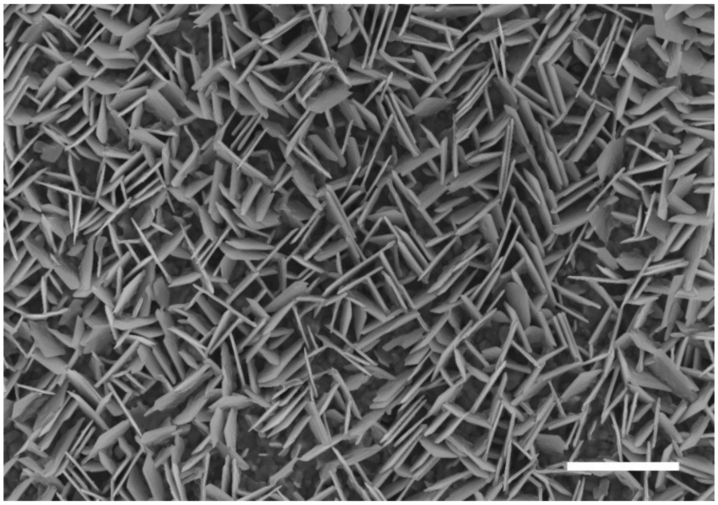

Supplementary Figure 1. SEM image of as-deposited SnNA. Scale bar, 2 μm.

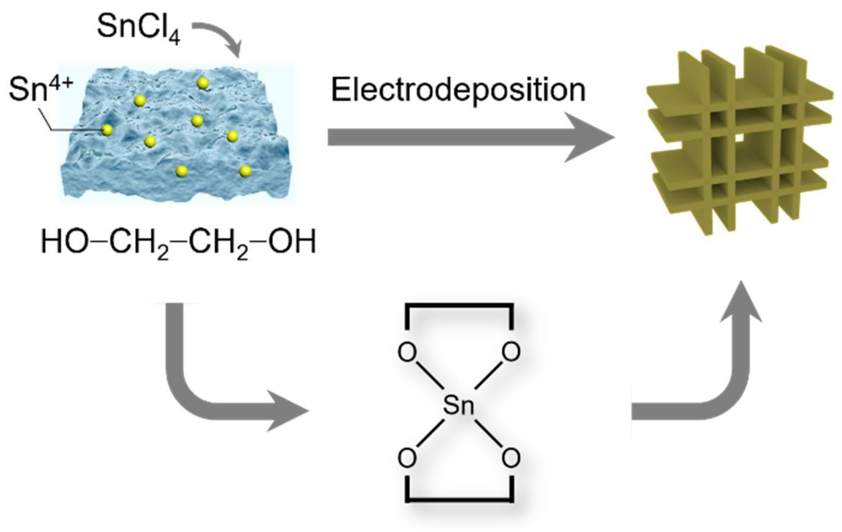

Supplementary Figure 2. Schematic illustration of the formation of Sn nanowalls at the assistant of glycol.

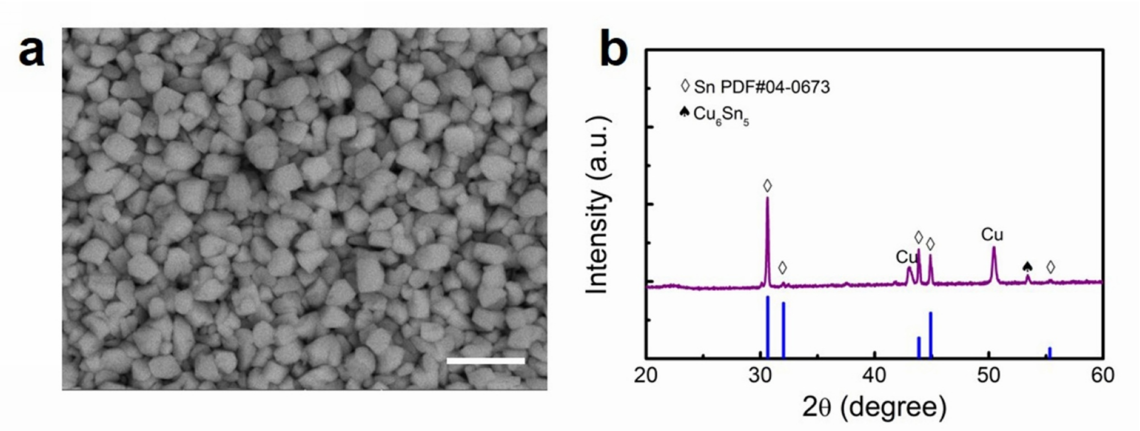

Supplementary Figure 3. Morphology and structure of Sn film electrodeposited from aqueous electrolyte of $SnCl_4$ without glycol. (a) SEM image, (b) XRD pattern. Scale bar, 2 μm.

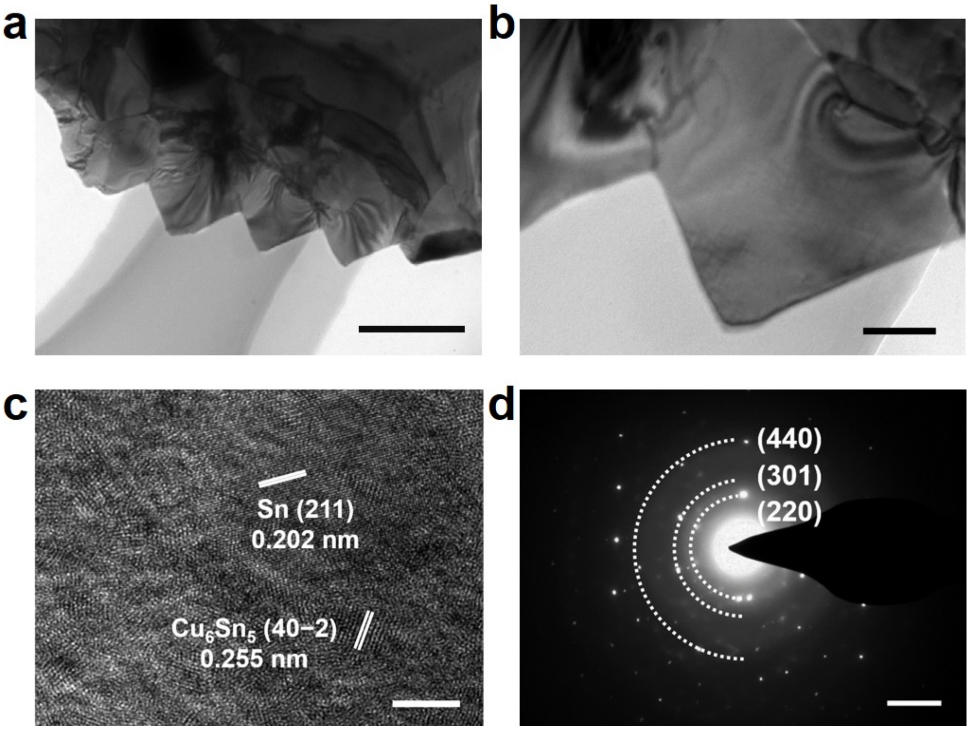

Supplementary Figure 4. Microscopic characterization of Sn nanowalls. (a, b) TEM images. (c) High-resolution TEM image shows lattice spacings of 0.202 nm and 0.255 nm, corresponding to the (211) plane of the tetragonal Sn and the (40-2) plane of Cu_6Sn_5 alloy, respectively. (d) Selected-area electron diffraction pattern. Scale bar, (a) 500 nm, (b) 100 nm, (c), 5 nm, (d) 5 1/nm.

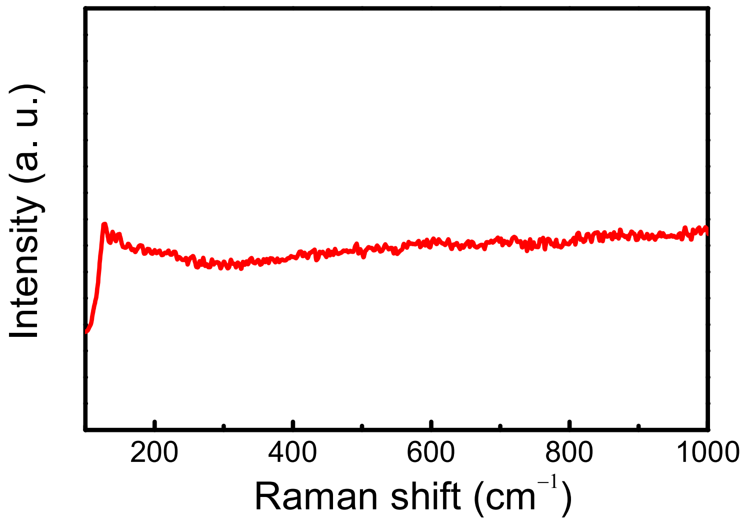

Supplementary Figure 5. Raman spectrum of annealed SnNA shows a featureless pattern.

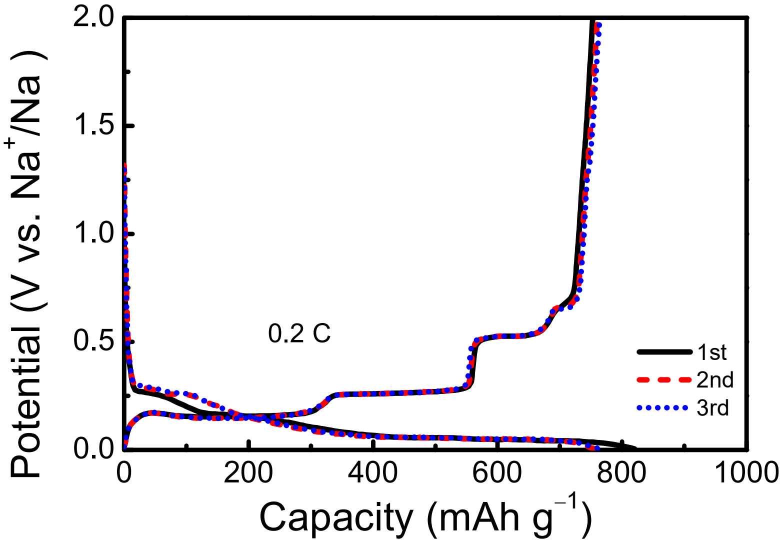

Supplementary Figure 6. Galvanostatic charge-discharge curves of SnNA annealed in Ar-H_2 gas flow, exhibiting an initial Coulombic efficiency of 92%.

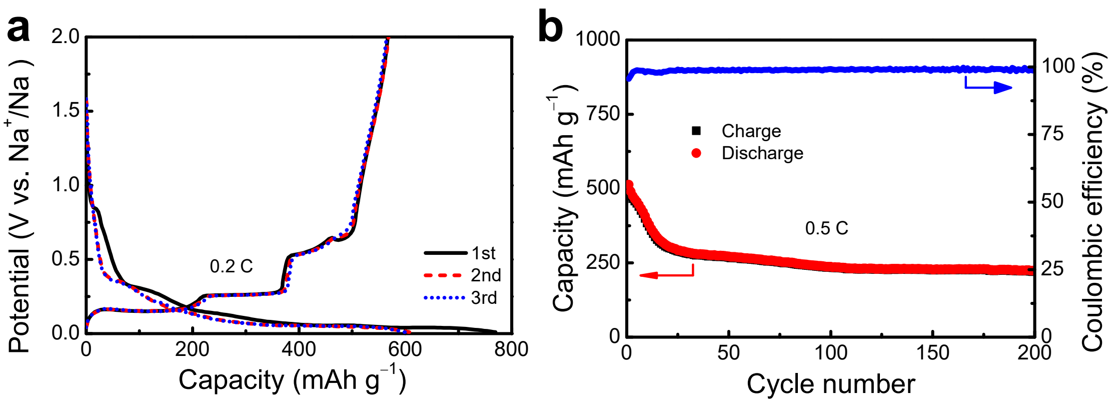

Supplementary Figure 7. Electrochemical sodium storage in Sn film electrode. (a) Galvanostatic curves at a rate of 0.2 C for initial cycles. (b) Cycling performance at a rate of 0.5 C for 200 cycles.

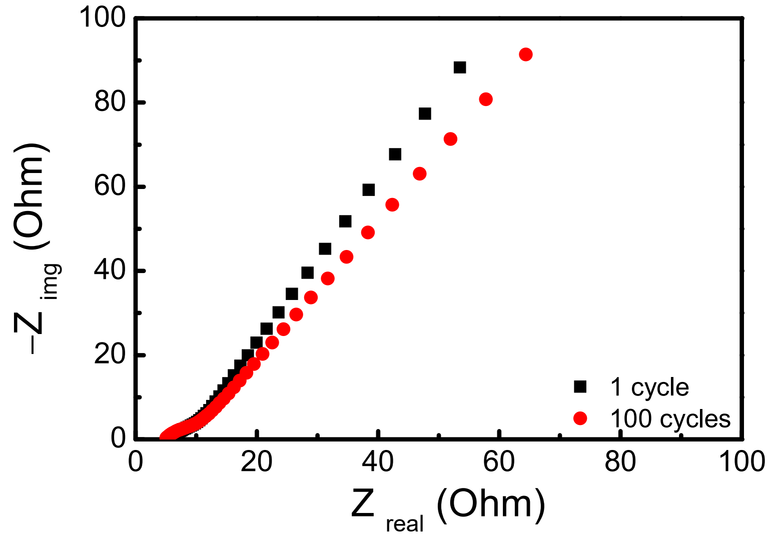

Supplementary Figure 8. Impedance spectra of a SnNA electrode after 1 cycle and 100 cycles.

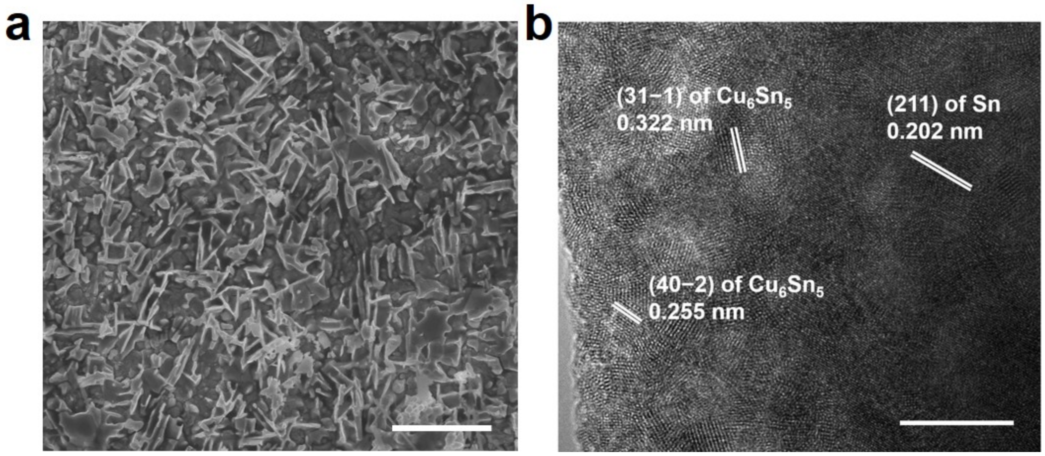

Supplementary Figure 9. Microscopic characterization of a SnNA electrode after 100 cycles. (a) SEM and (b) TEM images. Scale bar, (a) 4 μm, (b) 10 nm.

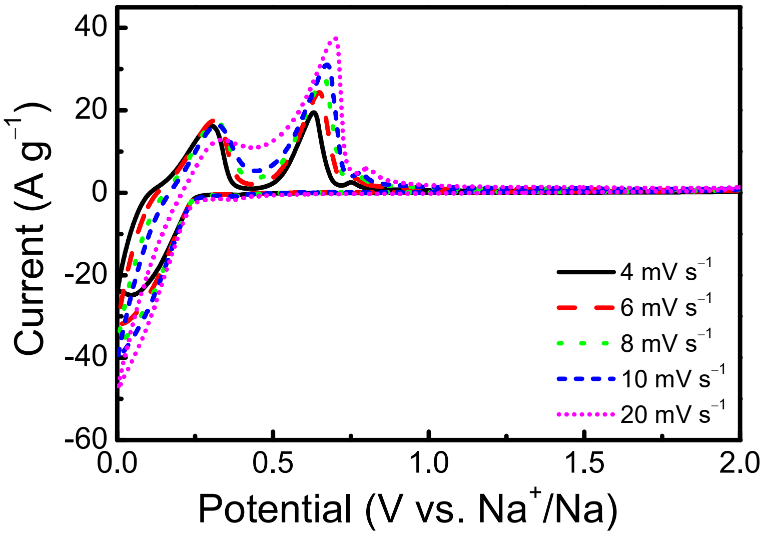

Supplementary Figure 10. CV curves of a SnNA electrode at sweep rates ranging from 4 to 20 mV s^{-1}.

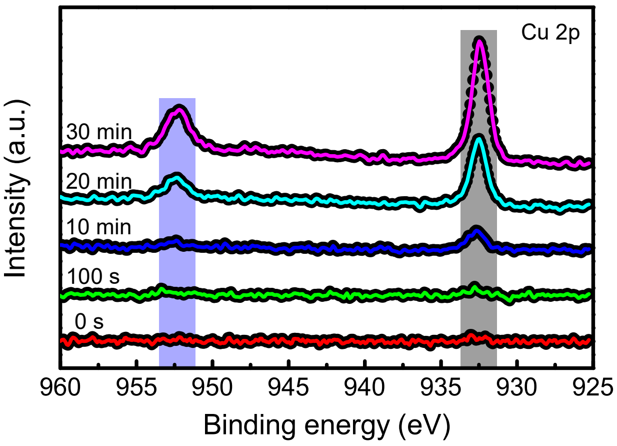

Supplementary Figure 11. XPS core-level spectra of Cu 2p for SnNA at various etching depths (durations), showing the continuous increase in the content of Cu from the top Sn to the bottom Cu.

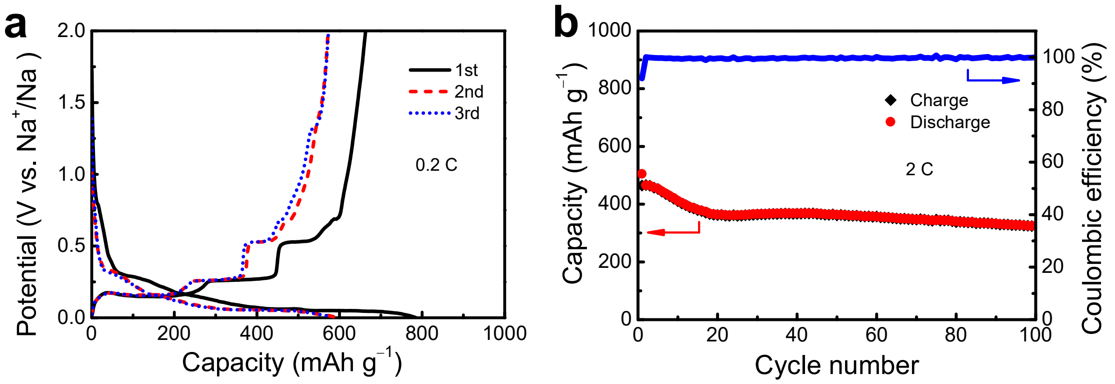

Supplementary Figure 12. Electrochemical sodium storage in SnNA without thermal annealing. (a) Galvanostatic curves at a rate of 0.2 C for initial cycles. (b) Cycling performance at a rate of 2 C for 100 cycles.

Supplementary Table 1. Comparison of electrochemical sodium storage in Sn-based anodes.

Sample	Capacity retention	Rate capacity	1st efficiency	Ref.
Sn dots@N-C	580 mAh g^{-1} for 300 cycles@0.5 A g^{-1}	483 mAh g^{-1} @2 A g^{-1}	70%	1
8-nm Sn@C	415 mAh g^{-1} for 500 cycles@1 A g^{-1}	349 mAh g^{-1} @4A g^{-1}	67%	2
Sn-TiO_2 @CNF	413 mAh g^{-1} for 400 cycles@0.1 A g^{-1}		62%	3
3D C/Sn/Ni/TMV1cys	405 mAh g^{-1} for 150 cycles@0.05 A g^{-1}		~60%	4
Sn@wood fiber	145 mAh g^{-1} for 1000 cycles@0.2 A g^{-1}	339 mAh g^{-1} @84 mA g^{-1}	23%	5
Sn@CNT	398 mAh g^{-1} for 150 cycles@0.1 A g^{-1}	117 mAh g^{-1} @2 A g^{-1}	46%	6
Sn@graphene	413 mAh g^{-1} for 100 cycles@0.1 A g^{-1}	106 mAh g^{-1} @3.2 A g^{-1}	~55%	7
[a] SnNA	501 mAh g^{-1} for 300 cycles@4.2 A g^{-1}	610 mAh g^{-1} @4.2 A g^{-1}	92%	This work

[a] Here desodiation capacity is adopted, whereas most others adopt sodiation capacities.

References

1. Liu, Y., Zhang, N., Jiao, L. & Chen, J. Tin nanodots encapsulated in porous nitrogen-doped carbon nanofibers as a free-standing anode for advanced sodium-ion batteries. *Adv. Mater.* **11**, 6702-6707 (2015).

2. Liu, Y., Zhang, N., Jiao, L., Tao, Z. & Chen, J. Ultrasmall Sn nanoparticles embedded in carbon as high-performance anode for sodium-ion batteries. *Adv. Funct. Mater.* **25**, 214-220 (2015).

3. Mao, M., et al. Pipe-wire TiO_2–Sn@carbon nanofibers paper anodes for lithium and sodium ion batteries. *Nano Lett.* **17**, 3830-3836 (2017).

4. Liu, Y., et al. Tin-coated viral nanoforests as sodium-ion battery anodes. *ACS Nano* **7**, 3627-3634 (2013).

5. Zhu, H., et al. Tin anode for sodium-ion batteries using natural wood fiber as a mechanical buffer and electrolyte reservoir. *Nano Lett.* **13**, 3093-3100 (2013).

6. Ruan, B., et al. Carbon-encapsulated Sn@N-doped carbon nanotubes as anode materials for application in SIBs. *ACS Appl. Mater. Interfaces* **9**, 37682-37693 (2017).

7. Luo, B., Qiu, T., Ye, D., Wang, L. & Zhi, L. Tin nanoparticles encapsulated in graphene backboned carbonaceous foams as high-performance anodes for lithium-ion and sodium-ion storage. *Nano Energy* **22**, 232-240 (2016).
